# Early discharge following colectomy for colon cancer: A national perspective

**DOI:** 10.1371/journal.pone.0294256

**Published:** 2024-02-16

**Authors:** Arjun Verma, Syed Shahyan Bakhtiyar, Konmal Ghazal Ali, Nikhil Chervu, Sara Sakowitz, Hanjoo Lee, Peyman Benharash

**Affiliations:** 1 Cardiovascular Outcomes Research Laboratories (CORELAB), David Geffen School of Medicine at UCLA, University of California Los Angeles, Los Angeles, California, United States of America; 2 Department of Surgery, David Geffen School of Medicine at UCLA, University of California Los Angeles, Los Angeles, California, United States of America; 3 Department of Surgery, University of Colorado Anschutz Medical Center, Aurora, Colorado, United States of America; 4 Division of Colon and Rectal Surgery, Department of Surgery, Harbor-UCLA Medical Center, Torrance, California, United States of America; 5 Division of Cardiac Surgery, Department of Surgery, David Geffen School of Medicine at UCLA, University of California Los Angeles, Los Angeles, California, United States of America; University of L’Aquila, ITALY

## Abstract

**Background:**

Although early discharge after colectomy has garnered significant interest, contemporary, large-scale analyses are lacking.

**Objective:**

The present study utilized a national cohort of patients undergoing colectomy to examine costs and readmissions following early discharge.

**Methods:**

All adults undergoing elective colectomy for primary colon cancer were identified in the 2016–2019 Nationwide Readmissions Database. Patients with perioperative complications or prolonged length of stay (>8 days) were excluded to enhance cohort homogeneity. Patients discharged by postoperative day 3 were classified as *Early*, and others as *Routine*. Entropy balancing and multivariable regression were used to assess the risk-adjusted association of early discharge with costs and non-elective readmissions. Importantly, we compared 90-day stroke rates to examine whether our results were influenced by preferential early discharge of healthier patients.

**Results:**

Of an estimated 153,996 patients, 45.5% comprised the *Early* cohort. Compared to *Routine*, the *Early* cohort was younger and more commonly male. Patients in the *Early* group more commonly underwent left-sided colectomy and laparoscopic operations. Following multivariable adjustment, expedited discharge was associated with a $4,500 reduction in costs as well as lower 30-day (adjusted odds ratio [AOR] 0.74, p<0.001) and 90-day non-elective readmissions (AOR 0.74, p<0.001). However, among those readmitted within 90 days, *Early* patients were more commonly readmitted for gastrointestinal conditions (45.8 vs 36.4%, p<0.001). Importantly, both cohorts had comparable 90-day stroke rates (2.2 vs 2.1%, p = 0.80).

**Conclusions:**

The present work represents the largest analysis of early discharge following colectomy for cancer and supports its relative safety and cost-effectiveness.

## Introduction

Colorectal cancers are the fourth most common malignancy in the world, with nearly 2 million new cases each year [1]. Patients with early stage disease most commonly undergo resection of the diseased segment and potential adjuvant chemoradiation [2]. Of note, advances in chemotherapeutic agents, surgical technique and perioperative care have contributed to declining mortality, complications and length of stay following colectomy for cancer [3]. However, hospitalization costs and unplanned readmission rates remain high and rising [3, 4].

Among several cost containment strategies, early discharge pathways have garnered significant interest across surgical specialties [5–8]. Early discharge following colectomy has been facilitated, in part, by Enhanced Recovery After Surgery (ERAS) protocols, which aim to reduce surgical stress, complications and length of stay [9]. While the relative safety of early discharge has been established in cardiothoracic and foregut surgery, concerns regarding increased readmissions remain for patients undergoing colectomy [5, 7, 8, 10]. Such apprehensions may stem from early reports of high readmissions among those discharged before postoperative day 4, as well as pragmatic limitations to patient monitoring following discharge [10]. Nonetheless, contemporary, national studies of early discharge after colectomy are lacking.

Therefore, the present study used a national database to evaluate the association between early discharge and outcomes following colectomy for cancer. We hypothesized that early discharge would be linked to decreased resource use at the index admission as well as at subsequent hospitalizations. We secondarily assessed the presence of center-level variation in early discharge, hypothesizing that high surgical volume and hospital teaching status would be associated with increased implementation of early discharge.

## Methods

This was a retrospective cohort study of the 2016–2019 Nationwide Readmissions Database (NRD). The NRD is the largest, all-payer readmissions database in the United States and is maintained by the Agency for Healthcare Research and Quality as part of the Healthcare Cost and Utilization Project. Through the application of survey weighting methodology, the NRD provides accurate estimates for approximately 60% of hospitalizations in the United States. Moreover, patients are tracked across hospitalizations within each state and calendar year using unique linkage numbers. Given the deidentified nature of the NRD, the Institutional Review Board at the University of California, Los Angeles deemed the present study exempt from full review and waived the requirement for informed consent.

**Fig 1:**
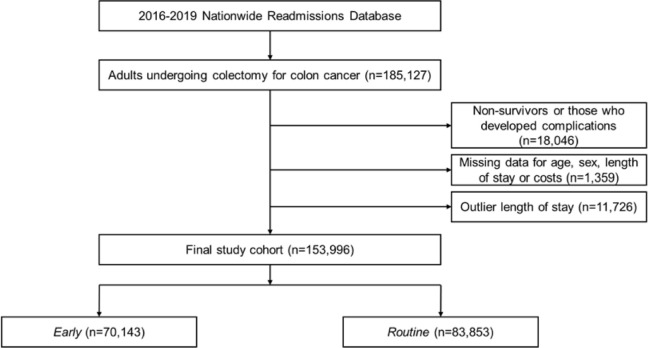
Study flow diagram.

All adults (≥18 years) undergoing elective colectomy (left-sided, right-sided and total) for primary colon cancer were identified using *International Classification of Diseases 10*^*th*^ *Revision* diagnosis and procedure codes. Those who developed major complications (ischemic/hemorrhagic stroke, ventricular tachycardia/fibrillation, cardiac arrest, tamponade, deep vein thrombosis, pulmonary embolism, pneumonia, prolonged mechanical ventilation, sepsis, acute kidney injury and hemorrhage) or did not survive to discharge, were excluded. Patients with length of stay >8 days (90^th^ percentile) were also not considered. Records with missing data for age, sex, length of stay (LOS) and costs were excluded (<1%). Based on previously published thresholds, patients were stratified *a priori* into *Early* (LOS≤3 days) and *Routine* (LOS>4 days) cohorts [4, 11, 12]. Derivation of the study population is shown in Fig 1.

Patient and hospital characteristics were defined in accordance with the NRD data dictionary [13]. The Van Walraven modification of the Elixhauser Comorbidity Index was used to quantify the burden of chronic conditions for each patient, while individual comorbidities and stoma creation were ascertained using ICD-10 codes [14]. Operative approach was stratified into open and minimally invasive (laparoscopic and robotic-assisted). Annual hospital volume was calculated as the total number of elective colonic resections for cancer performed each year at a given institution. Reasons for readmission were tabulated using Diagnosis Related Group codes. Hospitalization costs were generated through application of cost-to-charge ratios and inflation adjusted to the 2019 Personal Health Index [15].

The primary endpoints of the present study were index hospitalization costs and non-elective readmission at 30 and 90 days. We secondarily evaluated in-hospital mortality, reason for readmission, costs upon rehospitalization and combined costs (sum of index hospitalization and readmission costs). Importantly, we performed a negative control analysis to examine whether our results were influenced by preferential early discharge of patients with lower disease severity and greater functional status. For this analysis, we compared 90-day stroke rates between the *Early* and *Routine* cohorts, hypothesizing that stroke rates would be strongly correlated with disease severity but not early discharge status. Finally, we assessed inter-hospital variation in the utilization of early discharge.

Categorical variables are reported as frequencies and compared using the Pearson Chi-Squared test. Continuous variables are summarized as means with standard deviation and are compared across groups using the Adjusted Wald test. A multi-level model with early discharge as the dependent variable was developed, with patient characteristics as the first level and unique hospital identifier as the second. The intraclass correlation coefficient (ICC) was calculated to quantify the degree of observed variation in early discharge that was attributable to care at each center. In addition, the absolute values of random effects were generated and considered to be the hospital-specific, risk-adjusted rate of early discharge at each institution.

To adjust for significant differences in baseline characteristics between *Early* and *Routine* cohorts, we employed entropy balancing when analyzing outcomes following early discharge. While propensity matching techniques frequently rely on inadequate logistic regression models to generate propensity scores, entropy balancing employs mathematical techniques to achieve covariate balance. Its application in retrospective cohort studies has been extensively validated [16–18]. Following entropy balancing, multivariable linear regressions were used to assess the relationship between early discharge and costs at the index admission and upon rehospitalization. Logistic regression and Royston Parmar flexible modeling were used to quantify the association between early discharge status and non-elective readmissions. Variables were selected for inclusion into entropy balancing and risk-adjustment models using elastic net regularization [19]. This autonomous algorithm increases out-of-sample generalizability and reduces collinearity between covariates. Regression results are reported as adjusted odds ratios (AOR), β-coefficients and hazard ratios [HR], with 95% confidence intervals (CI). A p-value less than 0.05 was considered statistically significant. All statistical analysis was performed using Stata 16.0.

## Results

**Fig 2:**
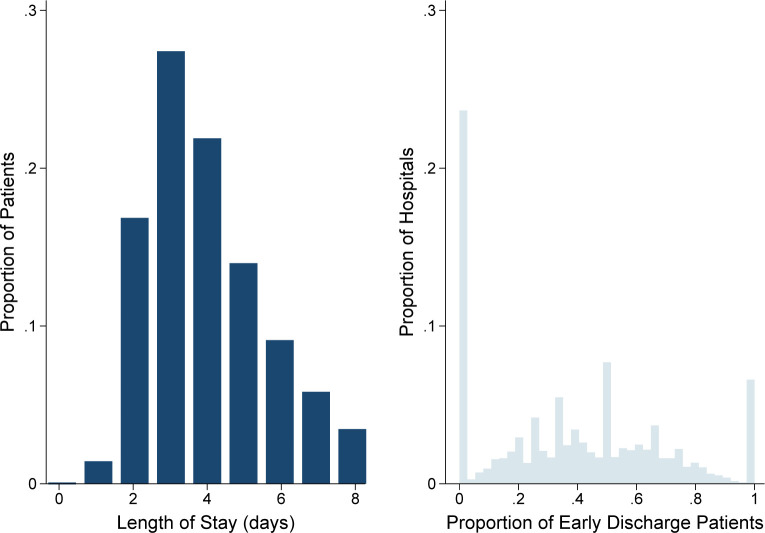
Distribution of patient length of stay and center-level utilization of early discharge.

Of an estimated 153,996 hospitalizations entailing colectomy for colon cancer (mean LOS 3.8 ± 1.5 days), 70,143 (45.5%) comprised the *Early* cohort (Fig 2A). Compared to *Routine*, *Early* patients were more commonly male (49.3 vs 47.5%, p<0.001), younger (65.0 ± 12.3 vs 67.6 ± 12.8 years, p<0.001) and had a lower burden of comorbidities as measured by the Elixhauser Comorbidity Index (2.9 ± 1.6 vs 3.5 ± 1.8, p<0.001). Specifically, early discharge patients had lower rates of congestive heart failure, coagulopathy and liver disease (Table 1). Compared to *Routine*, the *Early* cohort more frequently underwent left colectomy (34.4 vs 31.1%, p<0.001), but less commonly right (64.4 vs 66.4%, p<0.001) and total colectomy (1.2 vs 2.5%, p<0.001). Furthermore, the *Routine* cohort more often underwent open colectomy and stoma creation, relative to *Early* (Table 1).

**Table 1:** Comparison of *Early* and *Routine* cohorts. *SD*: *Standard Deviation*.

	*Early*	*Routine*	
	(n = 70,143)	(n = 83,853)	*P-value*
Age (years, mean ± SD)	65.0 ± 12.3	67.6 ± 12.8	<0.001
Female Sex (%)	50.7	52.5	<0.001
Elixhauser Comorbidity Index (mean ± SD)	2.9 ± 1.6	3.5 ± 1.8	<0.001
*Income quartile (%)*			<0.001
>75^th^ Percentile	25.3	19.7	
51^st^-75^th^ Percentile	26.8	24.8	
26^th^-50^th^ Percentile	26.2	28.5	
<26^th^ Percentile	21.7	27.0	
*Insurance coverage (%)*			<0.001
Private	41.2	30.4	
Medicare	51.4	60.5	
Medicaid	4.5	6.0	
Other Payer	2.9	3.2	
*Extent of Resection (%)*			<0.001
Left Sided Colectomy	34.4	31.1	
Right Sided Colectomy	64.4	66.4	
Total Colectomy	1.2	2.5	
Minimally Invasive Operation (%)	65.1	40.8	<0.001
Stoma Creation (%)	1.5	4.2	<0.001
*Comorbidities (%)*			
Congestive Heart Failure	3.8	6.5	<0.001
Coronary Artery Disease	11.5	14.8	<0.001
Valve Disease	2.9	4.2	<0.001
Pulmonary Circulation Disorders	0.8	1.5	<0.001
Peripheral Vascular Disease	3.8	4.8	<0.001
Hypertension	53.4	60.5	<0.001
Other Neurologic Disorders	1.6	2.5	<0.001
Chronic Pulmonary Disease	11.9	15.4	<0.001
Hypothyroidism	10.8	12.1	<0.001
Liver Disease	3.9	4.2	0.013
Coagulopathy	1.2	2.1	<0.001
Obesity	17.5	18.4	0.009
*Hospital teaching status (%)*			<0.001
Non-Metropolitan	6.2	1.2	
Metropolitan Non-Teaching	18.2	21.4	
Metropolitan Teaching	75.6	66.9	

On unadjusted comparison, the *Early* cohort accrued significantly lower costs at the index hospitalization ($15,700 ± 7,800 vs 19,300 ± 10,300, p<0.001). In addition, *Early* patients faced decreased rates of unplanned readmission at 30 (5.2 vs 7.9%, p<0.001) and 90 days (6.9 vs 10.3%, p<0.001) following discharge. Of note, *Early* patients were more commonly readmitted at 90 days for gastrointestinal reasons, including bleeding, surgical re-exploration and obstruction (45.8 vs 36.4%, p<0.001). However, among those readmitted within 90 days, in-hospital mortality, costs of the rehospitalization and combined costs were comparable between the *Early* and *Routine* cohorts (Table 2). Importantly, negative control analysis revealed comparable 90-day stroke rates (2.2 vs 2.1%, p = 0.80).

**Table 2:** Unadjusted comparison of resource utilization between *Early* and *Routine* cohorts. *SD*: *Standard Deviation*. *Costs are reported in $1*,*000s*.

	*Early*	*Routine*	
	(n = 70,143)	(n = 83,853)	*P-value*
Index Hospitalization Costs (mean ± SD)	15.7 ± 7.8	19.3 ± 10.3	<0.001
Unplanned Readmission (%)			
7 day	3.2	4.1	<0.001
30 day	5.2	7.9	<0.001
90 day	6.9	10.3	<0.001
Rehospitalization Costs (mean ± SD)			
7 day	15.7 ± 26.6	16.7 ± 25.6	0.29
30 day	14.6 ± 22.7	15.6 ± 22.9	0.13
90 day	14.7 ± 23.6	14.7 ± 20.9	0.93
Combined Hospitalization Costs (mean ± SD)			
7 day	31.2 ± 26.6	37.2 ± 30.0	<0.001
30 day	30.1 ± 24.7	36.3 ± 27.6	<0.001
90 day	30.2 ± 25.6	34.8 ± 25.3	<0.001

**Fig 3:**
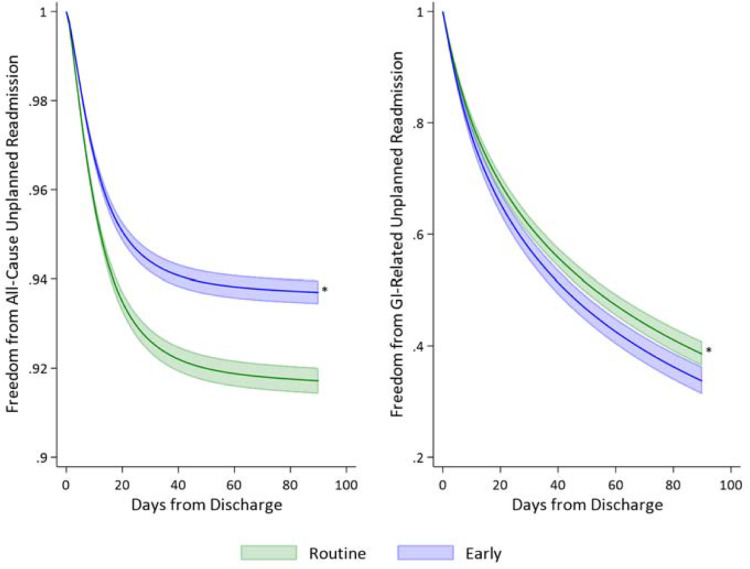
Royston Parmar of 90-day unplanned readmissions for any reason and for gastrointestinal (GI) conditions only.

Application of entropy balancing yielded adequately matched cohorts as demonstrated in S1 Fig. Following risk-adjustment using entropy balancing weights, early discharge was associated with a $3,400 decrement in index hospitalization costs (95% CI -3,700, -3,200). Moreover, the *Early* cohort faced reduced odds of 30- (AOR 0.74, 95% CI 0.69–0.79) and 90-day non-elective readmissions (AOR 0.74, 95% CI 0.70–0.79). Royston Parmar, time-adjusted analysis of readmission revealed similar findings, with early discharge being associated with a 27% reduction in relative hazard of 90-day readmissions (HR 0.73, 95% CI 0.69–0.77, Fig 3A). Interestingly, among those readmitted within 90 days, the *Early* cohort had significantly increased hazards of readmission for gastrointestinal reasons (HR 1.14, 95% CI 1.05–1.24, Fig 3B). Moreover, there were no statistically significant differences in mortality and costs between the *Early* and *Routine* cohorts upon rehospitalization. Combined hospitalization costs were lower among the *Early* cohort (Table 3).

**Table 3:** Risk-adjusted association between early discharge status and resource utilization, relative to routine discharge. *Costs are reported as β-coefficients in $1*,*000s*, *while readmissions are reported as adjusted odds ratios (AOR)*.

	*AOR/β*	*95% Confidence Interval*
Index Hospitalization Costs	-3.4	-3.7, -3.2
Unplanned Readmission		
7 day	0.86	0.79–0.94
30 day	0.74	0.69–0.79
90 day	0.74	0.70–0.79
Rehospitalization Costs		
7 day	-0.3	-2.3, +1.7
30 day	-0.9	-2.3, +0.5
90 day	-0.05	-1.2, +1.1
Combined Hospitalization Costs		
7 day	-4.6	-6.9, -2.4
30 day	-5.5	-7.2, -3.9
90 day	-4.1	-5.5, -2.8

**Fig 4:**
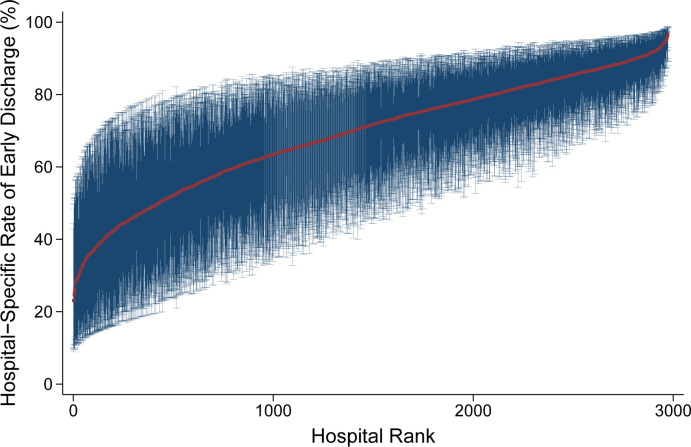
Center-level variation in early discharge following colectomy for colon cancer.

Of note, unadjusted, center-level rates of early discharge ranged from 0% to 100%, demonstrating significant interhospital variability (Fig 2B). Multi-level modeling also revealed significant variation in early discharge, with 20.9% of observed variation unexplained by patient factors (Fig 4). Centers in the highest decile of hospital-specific rates of early discharge had significantly increased annual surgical volume for colectomy (25 [17–40] vs 21 [14–36] cases/year, p<0.001). In addition, hospitals with high risk-adjusted rates of early discharge were more commonly teaching hospitals (72.8 vs 66.6%, p<0.001).

## Discussion

The present study examined the impact of early discharge on resource utilization following colectomy for cancer. After classification of patients into *Early* and *Routine* cohorts based on previously reported thresholds, we observed a significant decrement in hospitalization costs among those discharged before postoperative day 4. Moreover, early discharge was associated with reduced unplanned readmissions, but increased rates of rehospitalization specifically for gastrointestinal conditions. Finally, we noted the presence of significant center-level variation in the incidence of early discharge and found that high utilizing institutions were more commonly high volume, teaching hospitals. Several of our findings warrant further discussion.

Following risk-adjustment, the *Early* cohort accrued significantly reduced hospitalization costs at the index admission, relative to the *Routine* cohort. Specifically, early discharge was associated with a $3,400 decrement in episodic expenditure, which is most attributable to shortened length of stay. In agreement with our findings, prior studies of patients undergoing coronary artery bypass grafting and anatomic lung resection have noted cost-savings between $3,000 and $5,000 among those discharged early [7, 8]. Moon et al. analyzed the 2011–2017 NRD and found expedited discharge following colorectal resection to be associated with reduced costs at the index admission but greater costs upon rehospitalization [4]. In contrast, our analysis found no difference in the cost of readmission. This discrepancy may be explained by our examination of a more contemporary cohort comprised of exclusively colonic resections. Importantly, their analysis included perioperative complications at the index hospitalization, and therefore, may have been contaminated with outcomes of patients who were not suitable candidates for early discharge. Taken together with the existing literature, our findings suggest that early discharge is associated with a global reduction in costs.

Despite a growing body of literature demonstrating the safety of early discharge following cardiac, thoracic and pancreatic operations, concerns remain in the setting of colectomy [6]. In particular, early discharge may prevent timely identification of complications such as prolonged ileus, anastomotic breakdown and wound disruption. However, we found early discharge to be associated with significantly reduced rates of unplanned readmission at 7, 30 and 90 days, as well as unaltered mortality upon rehospitalization. To ensure that our findings were not related to functional status, we analyzed a falsified endpoint and determined that 90-day stroke rates were equivalent between both cohorts. This indicates that the lower readmissions faced by the *Early* cohort are unlikely to be attributable to selection bias relating to disease severity. The reduction in readmission rates may be due to the application of specific elements of ERAS protocols, such as early mobilization and feeding, which have been associated with enhanced recovery following major inpatient operations [20–23]. Nonetheless, compared to *Routine*, *Early* patients were more commonly readmitted for gastrointestinal conditions, including bleeding and reoperation. Our findings suggest that a subset of patients discharged early remain at risk for procedure-related complications and may not be suitable candidates. Prospective and mechanistic studies are necessary to identify factors associated with the development of major complications following early discharge.

In the present work, we found evidence of significant center-level variation in the incidence of early discharge. Of note, nearly 25% of hospitals did not discharge a single patient before postoperative day 4, while approximately 10% of institutions discharged all patients by postoperative day 3. Centers with a high proportion of patients undergoing expedited discharge had higher surgical volume and were more commonly teaching institutions, compared to other hospitals. Our findings are in agreement with a large body of literature demonstrating that variations in surgical practice and outcomes exist and are correlated with surgical volume and teaching status [7, 8, 21, 24–26]. The observed association between operative caseload and implementation of early discharge may be, in part, attributable to well-developed and streamlined care pathways at high volume centers. Moreover, teaching hospitals may have the resources and specialized staff needed to deliver appropriate counseling and information for outpatient recovery [9, 25, 27, 28]. Of note, high volume, teaching hospitals may be more likely to ensure patient and provider compliance with various elements of ERAS protocols, which, in turn, confers greater likelihood of early discharge and positive outcomes [9, 20, 21]. Future studies should identify factors associated with low adherence to early discharge pathways and disseminate quality improvement strategies to low utilizing centers.

This study has several limitations, including those inherent to its retrospective design. First, the NRD does not contain granular clinical information, including laboratory values, imaging studies and oncologic stage. Furthermore, we do not have access to the timing of first oral feeding, flatus or bowel movement. Although we attempted to control for selection bias through application of entropy balancing, multivariable adjustment and examination of a falsified endpoint, it is possible that our findings are due to the preferential discharge of healthy patients. The data source does not provide information about extraneous factors that may influence the likelihood of early discharge, including patients’ specific insurance plans and support systems outside of the hospital. Similarly, we cannot analyze the implementation of specific ERAS protocols, given the administrative nature of the NRD.

## Conclusions

The present study examined a national cohort of patients undergoing colectomy for cancer and used robust statistical methods to demonstrate reduced costs and readmissions among those discharged by the third postoperative day. In addition, we noted the presence of significant center-level variation in the implementation of early discharge. Our findings suggest that early discharge is safe and cost-effective, although further work is necessary to increase penetrance beyond high volume, teaching hospitals.

## Supporting information

S1 Checklist STROBE statement—checklist of items that should be included in reports of *cohort studies*. (DOC)

S1 Fig Standardized mean differences of patient characteristics before (blue circle) and after (yellow diamond) entropy balancing. (EPS)
